# Application of predonation hydration for vasovagal reactions during blood donation: a bibliometric analysis (2004–2023)

**DOI:** 10.3389/fmed.2025.1599519

**Published:** 2025-11-06

**Authors:** Cong Wang, Li Chen, Xiaomin Niu, Wenwen Shi, Xiaojing Liu, Meilin Li, Lihua Li, Wence Li

**Affiliations:** 1 Hebei Province Blood Center, Blood Security Department, Shijiazhuang, China; 2 Beijing Tongzhou Central Blood Station, Blood Security Department, Beijing, China

**Keywords:** predonation hydration, VVR, voluntary blood donation, VOSviewer, bibliometrix, CiteSpace

## Abstract

**Background:**

Vasovagal reaction (VVR) is one of the prevalent adverse reactions encountered during voluntary blood donation, posing a risk to subsequent donations by the same individuals. Here, we present a bibliometric study aimed at mitigating VVRs.

**Methods:**

This study retrieved pertinent publications on the correlation between predonation hydration and vasovagal reactions from the Web of Science Core Collection (WoSCC) database from 2004 to 2023. Bibliometric analysis was conducted employing bibliometrix R, VOSviewer, and CiteSpace.

**Results:**

This study encompassed 241 papers across 31 countries and 387 research institutions. Notably, the University of Bucharest (Romania, *n* = 12, 4.9%), the Australian Red Cross (Australia, *n* = 10, 4.1%), the University of Queensland (Australia, *n* = 10, 4.1%), Wayne State University (USA, *n* = 6, 2.5%), and Ohio State University (USA, *n* = 5, 2.1%) emerged as the top five contributors in publication output. The journal *Transfusion* was the most prolific publication venue in this field. Authors Thijsen, Amanda, and Deju, R. were identified as the most prolific contributors in this area. Over the past 3 years, research trends have predominantly focused on fear, complications, management, and hydration.

**Conclusion:**

This marks the first comprehensive overview of bibliometric research aimed at understanding the preventive and mitigative effects of predonation hydration on VVR. Predonation hydration as a strategy to prevent or alleviate VVR is currently the focal point of research interest. Our findings suggest that predonation hydration is a promising, low-cost approach to enhance donor safety and retention, although standardization of protocols is needed.

## Introduction

Blood transfusion is a life-saving intervention that heavily relies on voluntary blood donations from healthy individuals (1–3). The World Health Organization (WHO) regards voluntary blood donors as the cornerstone of a safe and sufficient blood supply. Recruiting more new volunteers to join the ranks of voluntary blood donors is important for ensuring the availability of clinical blood (4–7). Research suggests that many new blood donors may cease donating due to discomfort during the process, such as the vasovagal reaction (VVR) and fainting (8–13).

VVR refers to adverse reactions primarily exhibited throughout the body during blood donation, triggered by psychological factors (such as fear of needles or blood, anxiety) and physiological factors (such as lack of sleep or being underweight) among donors (14–16). Symptoms include general discomfort, weakness, pallor, sweating, anxiety, dizziness, and nausea. In severe cases, temporary loss of consciousness (fainting), convulsions, or loss of bladder or bowel control may occur. Fainting and falling can result in accidental injury (17–22). Young blood donors, women, first-time donors, and donors with estimated small blood volumes are most prone to VVR during blood donation (23–26). Studies indicate that after experiencing VVR, donors’ willingness to donate blood again decreases by 30%, and even mild VVR significantly reduces donors’ willingness to donate blood again (27).

Therefore, various strategies have been employed to reduce the incidence of VVR, including muscle relaxation and contraction exercises, psychological therapy, distraction therapy, and predonation hydration (1, 2, 6). A meta-analysis by Fisher et al. found that predonation hydration significantly reduces the incidence and severity of VVR (28). However, some studies have found no benefit of predonation hydration in preventing fainting and pre-fainting symptoms (29). Similarly, scholars have further pointed out that experiments have shown no difference in the incidence of VVR between consuming 330 or 500 mm of water before donation (30). Thus, there is a need for an objective method to scientifically evaluate the relationship between predonation hydration and VVR occurrence.

Bibliometrics is a discipline that focuses on the literature and its quantitative characteristics, using mathematical and statistical approaches to study the distribution structure, quantitative relationships, changes, and quantitative management of literature information. It further explores certain structures, characteristics, and laws of science and technology (31, 32). Currently, there is no bibliometric study on the relationship between predonation hydration and VVR. We selected the 2004–2023 timeframe because 2004 marks the earliest year in which studies explicitly investigating predonation hydration and vasovagal reactions were indexed in the Web of Science Core Collection. This 20-year span encompasses the foundational randomized controlled trials, subsequent observational studies, and recent systematic reviews that together define the modern evidence base for this topic. By covering two decades, we were able to analyze both the emergence of the field and its more recent acceleration. We chose a bibliometric approach rather than a meta-analysis because our primary objective was to map the global research landscape—examining productivity by country and institution, collaboration networks, influential authors and journals, and evolving thematic trends. Meta-analysis is most appropriate when multiple studies share highly comparable designs, populations, interventions, and outcome definitions, enabling statistical pooling of results. In contrast, research on predonation hydration and VVR shows substantial heterogeneity in donor demographics, hydration volume, timing, outcome measurement, and severity grading. This variability currently limits the feasibility and interpretability of a pooled effect estimate. A bibliometric analysis offers a complementary advantage: it can identify research gaps, methodological inconsistencies, and collaboration patterns that inform the design of more standardized trials, which in turn will enable future meta-analyses. This study aims to conduct a bibliometric analysis of the development of predonation hydration and VVR over the past 20 years (2004 to 2023), providing more detailed and objective data support for scholars entering this field.

## Methods

### Searching strategy

We conducted a literature search on January 1, 2023, using the Web of Science Core Collection, selecting the period from January 1, 2004, to December 31, 2023, because 2004 marks the first year indexed studies directly linking predonation hydration to vasovagal reactions were published. This timeframe allows the capture of early foundational research and subsequent developments over two decades. The formula for searching was: (((TS = (Predonation hydration)) OR TS = (water)) AND TS = (vasovagal reactions)) OR TS = (VVR), with document types restricted to “articles” and “reviews,” while excluding letters, conference proceedings, and book chapters, among others. In addition to screening by document type, we further categorized the included studies by research design (randomized controlled trials, cluster randomized trials, prospective and retrospective observational studies, cross-sectional surveys, and systematic reviews/meta-analyses). This classification was performed manually based on study descriptions in titles, abstracts, or full texts, allowing us to qualify the level of evidence represented in the bibliometric dataset. The research period spanned from January 1, 2004, to December 31, 2023. The language was set to English. The final search results were saved in TXT format, comprising all records and cited references.

### Data analysis

The software used for bibliometric analysis primarily includes bibliometrix R (version 4.0.0), VOSviewer (version 1.6.20), and CiteSpace (version 6.2.R6 Advanced Edition).

In this study, “cooperation among countries” was defined as the presence of at least one publication in which authors from institutions in different countries collaborated, as indicated by their affiliations in the author list.

Bibliometrix R (version 4.0.0) is an online bibliometric analysis software accessed through RStudio (32). This R package is mainly utilized for quantitative analysis. With this software, our primary tasks involved generating publication networks across countries, topic trending, and three-domain maps.

VOSviewer (version 1.6.20) is a bibliometric visualization software capable of extracting key information from numerous publications and presenting it visually (31). In this study, VOSviewer was employed to analyze countries and institutions, journals and co-cited journals, authors and co-cited authors, as well as keyword co-occurrence.

CiteSpace (version 6.2.R6 Advanced Edition), developed by Professor Chaomei Chen, is another software for bibliometric analysis and visualization (33). This study used CiteSpace to produce dual-map overlays of journals and burst maps of references. In this study, a “publication burst” refers to a period when a particular reference or keyword experiences a sharp increase in citations over a short time, as identified using CiteSpace algorithms. Such bursts indicate emerging or shifting research interest and can point to novel strategies or influential studies relevant to clinical practice. For example, a burst in hydration-related keywords may reflect increased focus on this preventive strategy in donor management. Similarly, “keyword bursts” highlight topics gaining rapid attention, potentially signaling changes in clinical guidelines or preventive measures. Additionally, we conducted a quantitative analysis of publications using Microsoft Office Excel 2019 and created charts of publication output. The impact factors of all journals were sourced from the 2023 Journal Citation Reports (JCR).

## Results

### Analysis of annual publication output

**Figure 1:**
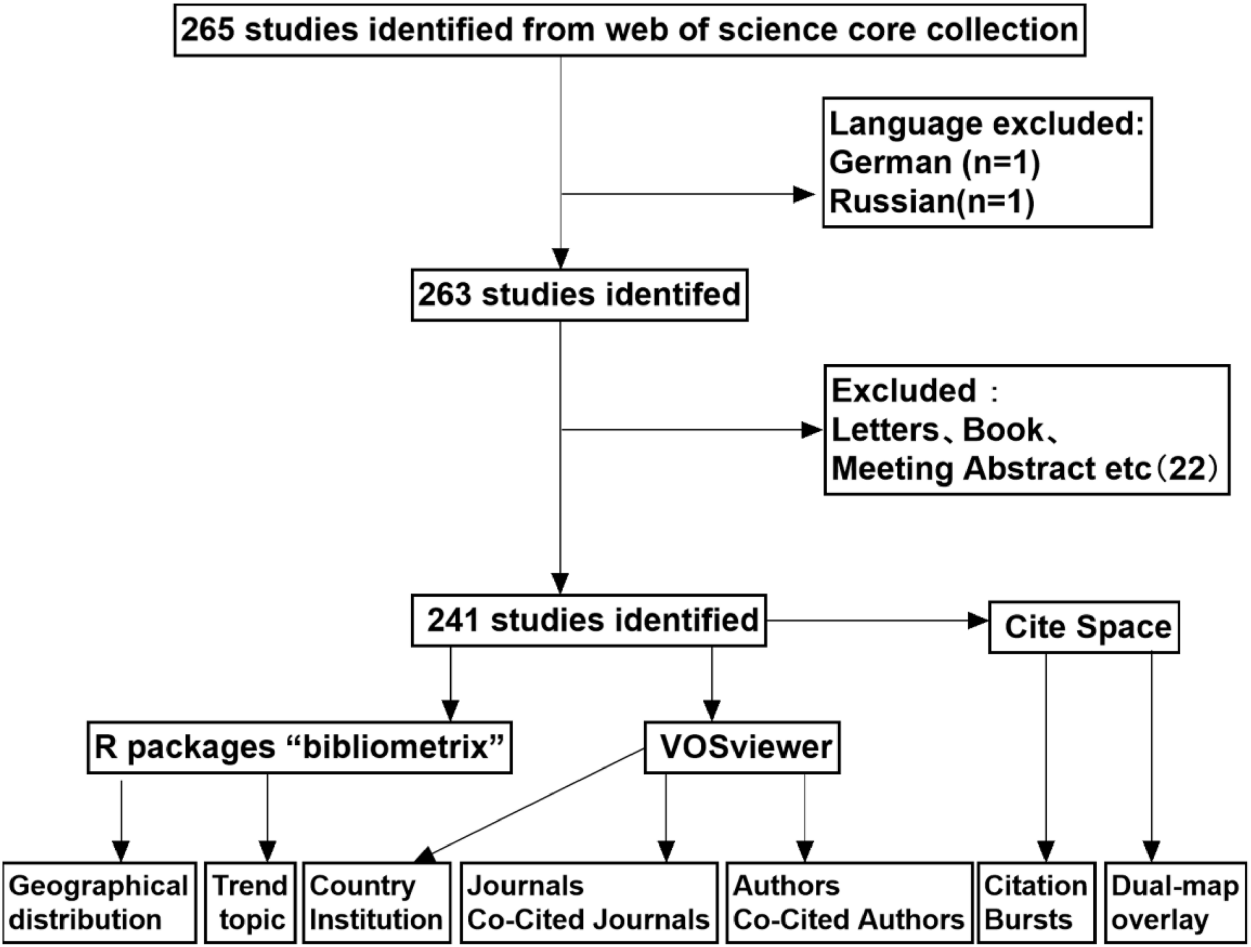
Publications screening flowchart.

**Figure 2:**
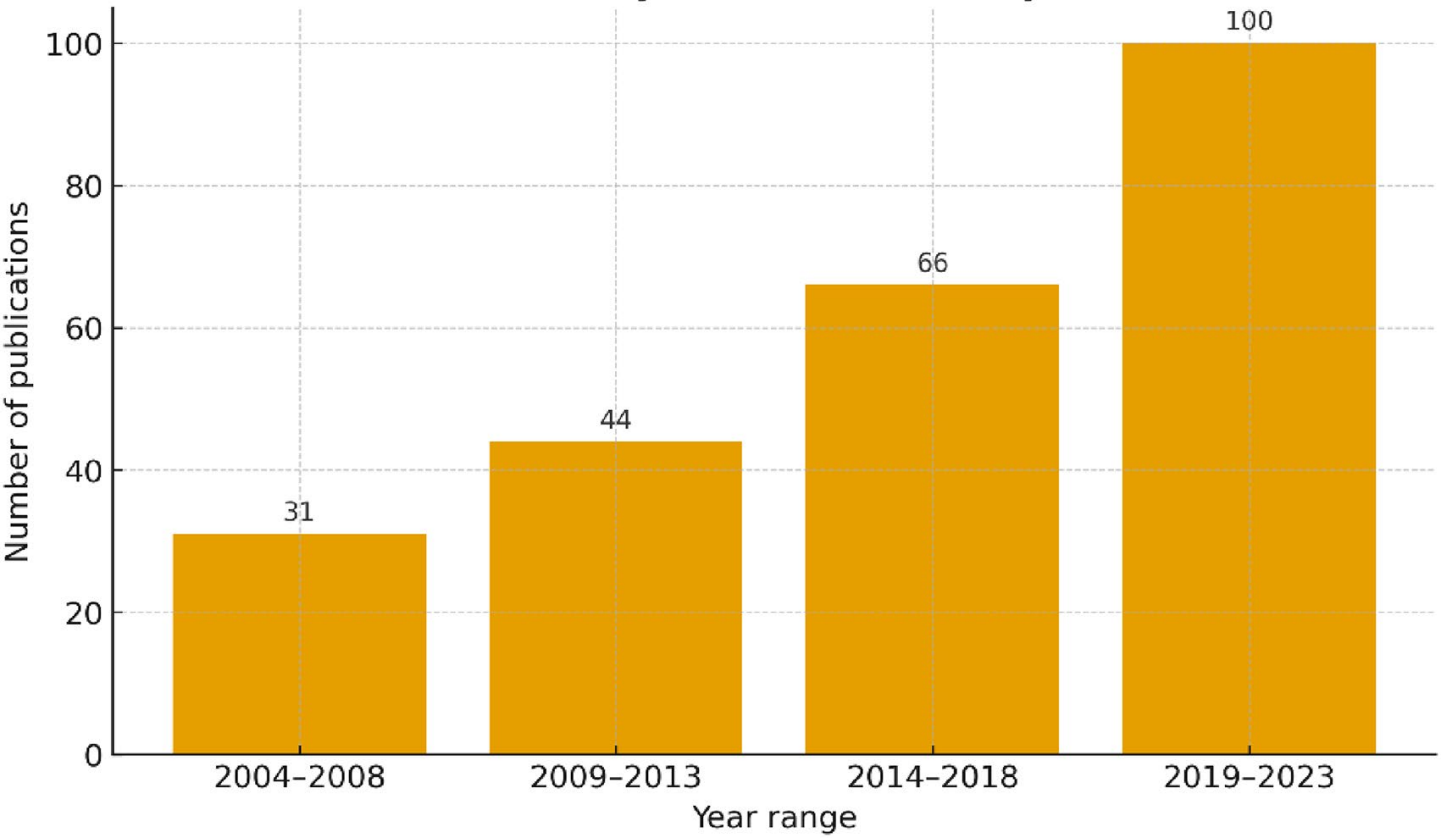
Annual output of research about predonation hydration and VVR.

With a predefined search strategy, a total of 265 studies on predonation hydration reducing the occurrence of VVR were identified between 2004 and 2023. After excluding 1 German and 1 Russian papers, 263 papers were retrieved. Subsequently, after excluding letters, conference papers, etc., a total of 241 papers, including “articles” and “reviews,” were finally included (Figure 1). From 2004 to 2014, the annual publication output steadily increased to around 8 articles. From 2015 to 2023, there was a rapid growth in annual publication output, with the number of papers reaching around average 17 per year, more than double that of the previous period. The year 2021 had the highest publication output, with 27 papers (Figure 2).

Among the 241 included publications, 34 (14.1%) were randomized controlled or cluster-randomized trials, 92 (38.2%) were observational studies (including prospective, retrospective, and cross-sectional designs), 21 (8.7%) were systematic reviews or meta-analyses, and the remaining 94 (39.0%) were narrative reviews or descriptive reports.

### Analysis of countries and institutions

**Figure 3:**
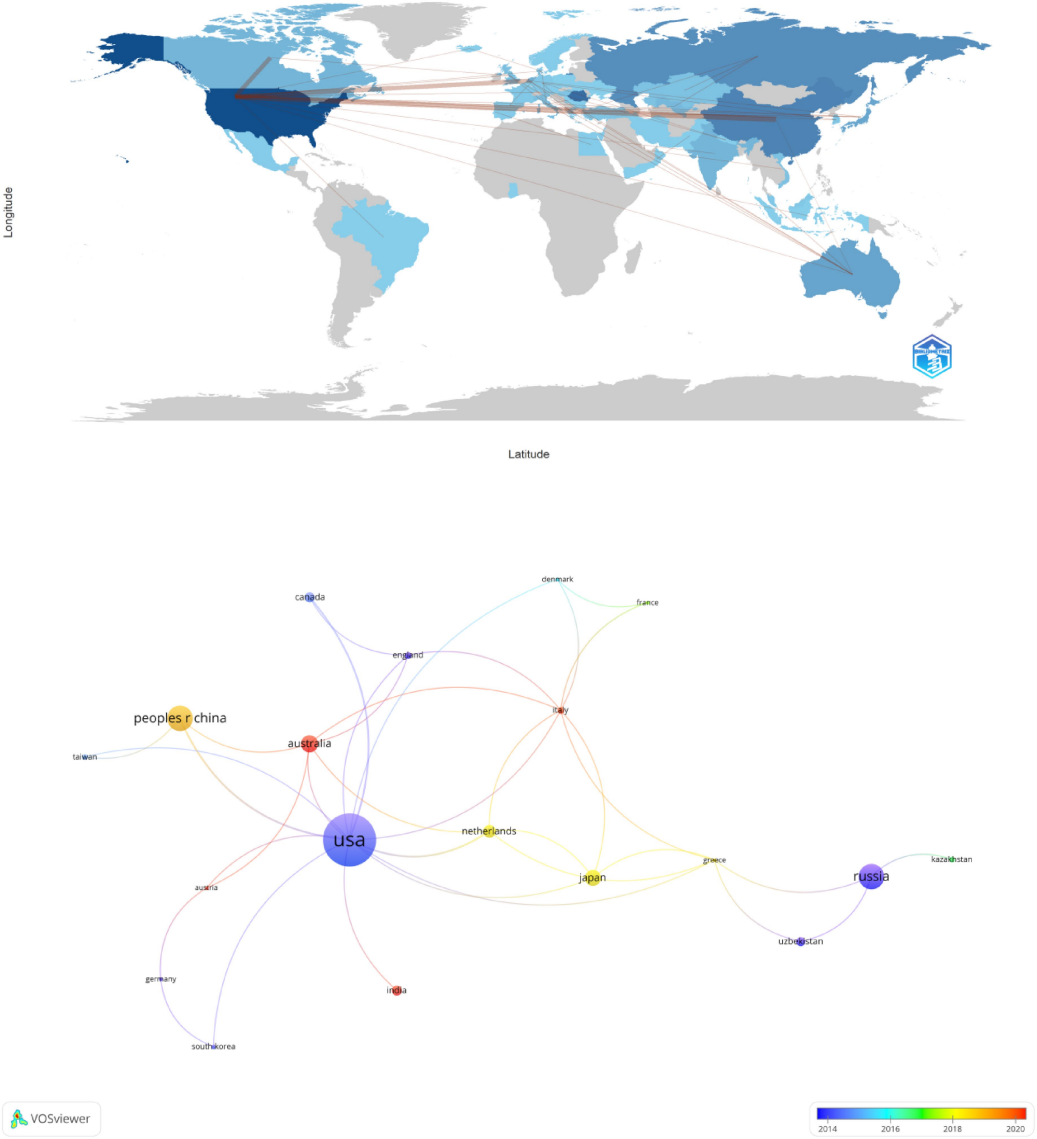
The geographical distribution and visualization of countries on research of predonation hydration and VVR.

The studies were primarily conducted in 31 countries and 387 research institutions. The top 5 countries in terms of publication output were the United States (*n* = 46, 19%), Romania (*n* = 34, 14%), China (*n* = 22, 9.1%), Russia (*n* = 22, 9.1%), and Australia (*n* = 15, 6.2%) (Table 1). The publication output of these 5 countries accounted for over half of the total publication output (57.4%). Using VOSviewer, we conducted a visualization of the downloaded data, analyzing the 31 countries based on publications equal to or greater than 2 and constructing a national publication network based on the quantity and relationship of publications for each country (Figure 3). We found active collaboration among different countries, as defined by co-authored publications between institutions from at least two different countries. For example, our network mapping showed strong cooperation between the United States, China, and India, indicated by multiple co-authored papers and high link strength values. Additionally, we observed that recent research outcomes are mainly concentrated in Australia, India, and Italy.

**Table 1:** Top5 countries and institutions on research of predonation hydration and VVR.

Rank	Country	Counts	Institution	Counts
1	The United States (North America)	46 (19%)	University of Bucharest (Romania)	12 (4.9%)
2	Romania (Europe)	34 (14%)	Australia Red Cross (Australia)	10 (4.1%)
3	China (Asia)	22 (9.1%)	The University of Queensland (Australia)	10 (4.1%)
4	Russia (Europe)	22 (9.1%)	Wayne State University (America)	6 (2.5%)
5	Australia (Europe)	15 (6.2%)	Ohio State University (America)	5 (2.1%)

The top 5 institutions in terms of publication output were the University of Bucharest (Romania, *n* = 12, 4.9%), Australian Red Cross (Australia, *n* = 10, 4.1%), University of Queensland (Australia, *n* = 10, 4.1%), Wayne State University (USA, *n* = 6, 2.5%), and Ohio State University (USA, *n* = 5, 2.1%) (Table 1). The publication output of these 5 institutions accounted for 17.7% of the total publication output.

### Journal analysis

A total of 136 journals have published papers relevant to this field. *Transfusion* had the highest publication output (*n* = 26, 10.8%), followed by *Transfusion and Apheresis Science* (*n* = 5, 2.1%), *Vox Sanguinis* (*n* = 5, 2.1%), and *Transfusion Medicine* (*n* = 5, 2.1%). Among the top five journals in terms of publication output, *Transfusion* had the highest impact factor (IF = 2.9), followed by *Voxs Sanguinis* (IF = 2.7) (Table 2).

**Table 2:** Top 5 journals and co-cited journals for research of predonation hydration and VVR.

Rank	Journal	Counts	JCR	IF	Co-cited Journal	Counts	JCR	IF
1	Transfusion	26	3	2.9	Transfusion	744	3	2.9
2	Transfusion and apheresis science	5	4	1.9	Transfusion and apheresis science	87	4	1.9
3	Transfusion medicine	5	4	1.5	Vox sanguinis	84	3	2.7
4	Vox sanguinis	5	3	2.7	Circulation	83	1	37.8
5	Transfusion clinique et biologique	3	4	1.7	Transfusion medicine	51	4	1.5

**Figure 4:**
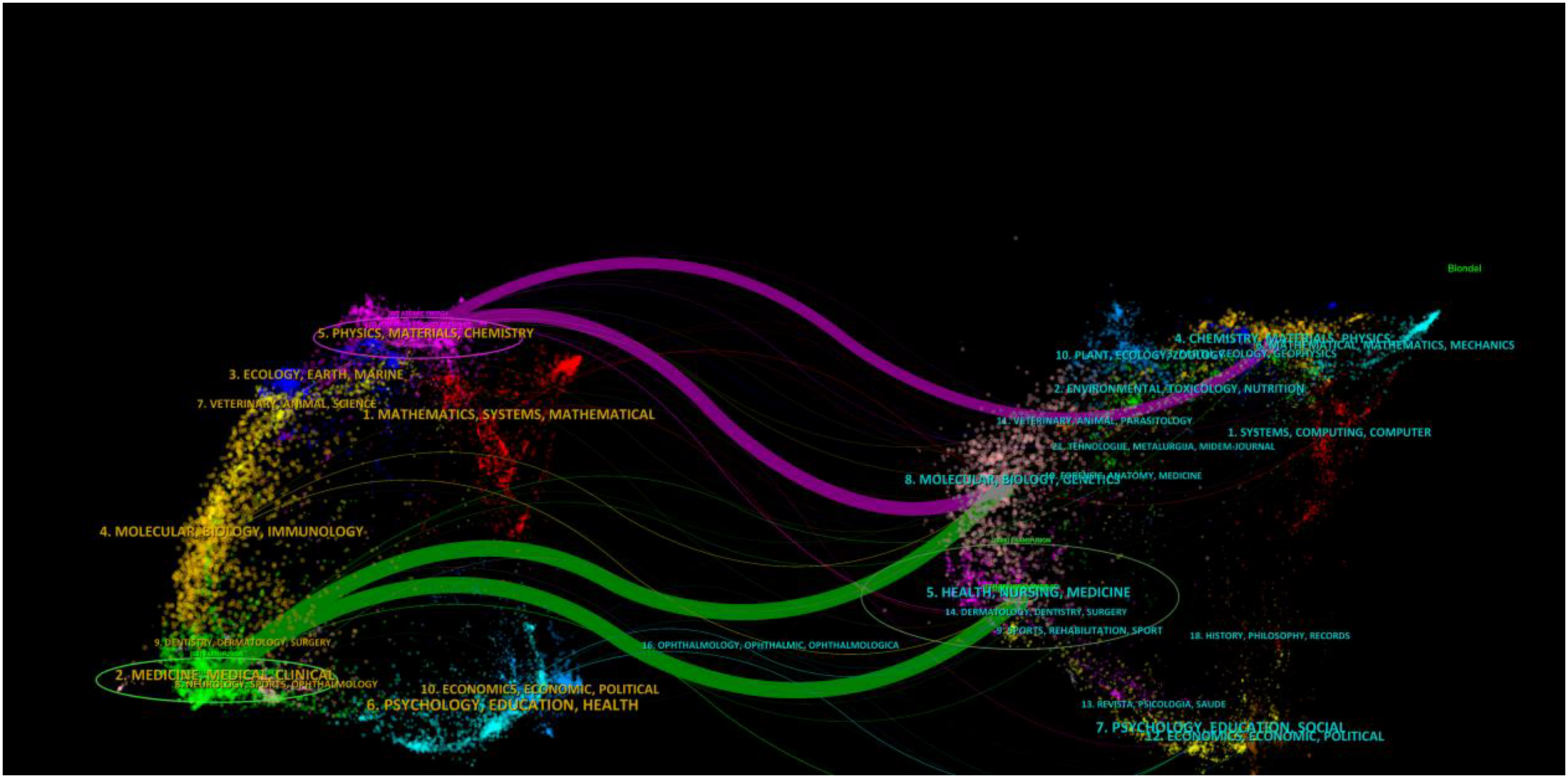
The dual-map overlay of journals on research of predonation hydration and VVR.

In the analysis of total citations to journals, *Transfusion* was the most frequently cited journal, with a total of 744 citations, followed by *Transfusion and Apheresis Science* with 87 citations, *Vox Sanguinis* with 84 citations, and *Circulation* with 83 citations. *Transfusion Medicine* had a total of 51 citations. Among the cited journals, *Circulation* had the highest impact factor (IF = 37.8), followed by *Transfusion* with an impact factor of (IF = 2.9) (Table 2). The overlay map illustrates the citation relationship between journals and co-cited journals, with citing journals on the left and cited journals on the right. As shown in Figure 4, the green paths represent the main citation paths (commonly known as the *Z* score). Through this map, we can clearly observe that papers published in health/nursing/medical journals are primarily cited by medical/health/clinical journals.

### Analysis of authors and co-cited authors

**Figure 5:**
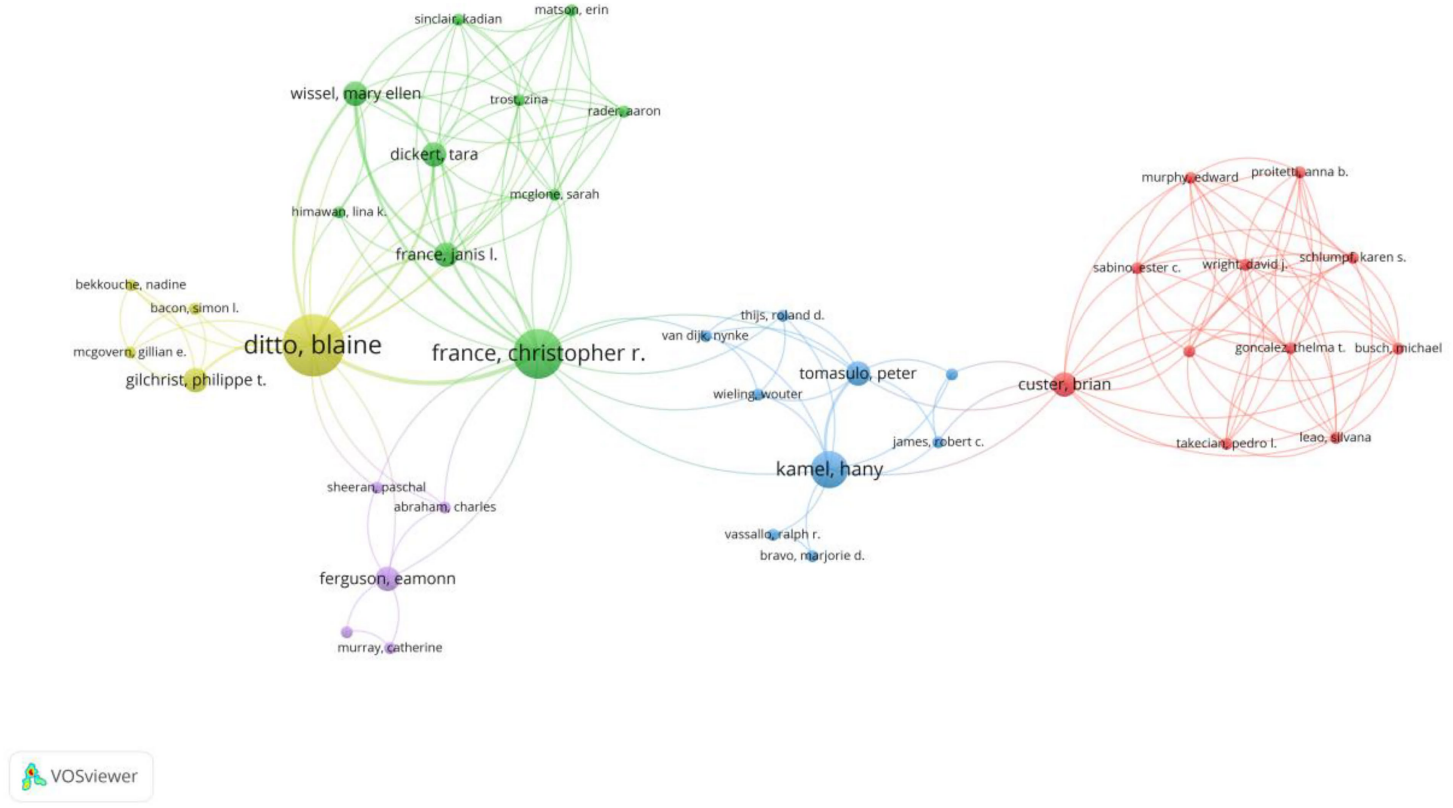
The visualization of authors on research of predonation hydration and VVR.

In this bibliometric study, a total of 1,013 authors contributed to VVR research. Among the top 5 authors with the highest publication output, Thijsen, Amanda, and Deju, R. were the most prolific authors, each having published 9 papers over 20 years (Table 3). Davison, Tanya E., and Masser, Barbara each had 8 publications. Utilizing VOSviewer to analyze the downloaded data, we constructed a visual authorship map based on authors who had published at least one paper. The size of the nodes indicates the number of papers authored by each individual, while the colors reflect different clusters of authors based on collaboration strength. A total of 5 clusters were identified (Figure 5).

**Table 3:** Top 5 authors and co-cited authors on research of predonation hydration and VVR.

Rank	Authors	Counts	Link strength	Co-cited authors	Counts	Link strength
1	thijsen, amanda	9	33	france, cr	147	874
2	deju, r.	9	24	ditto, b	97	755
3	masser, barbara	8	30	newman, bh	87	511
4	davison, tanya e.	8	28	eder, af	55	397
5	gurau, d.	7	21	thijsen, a	50	315

**Figure 6:**
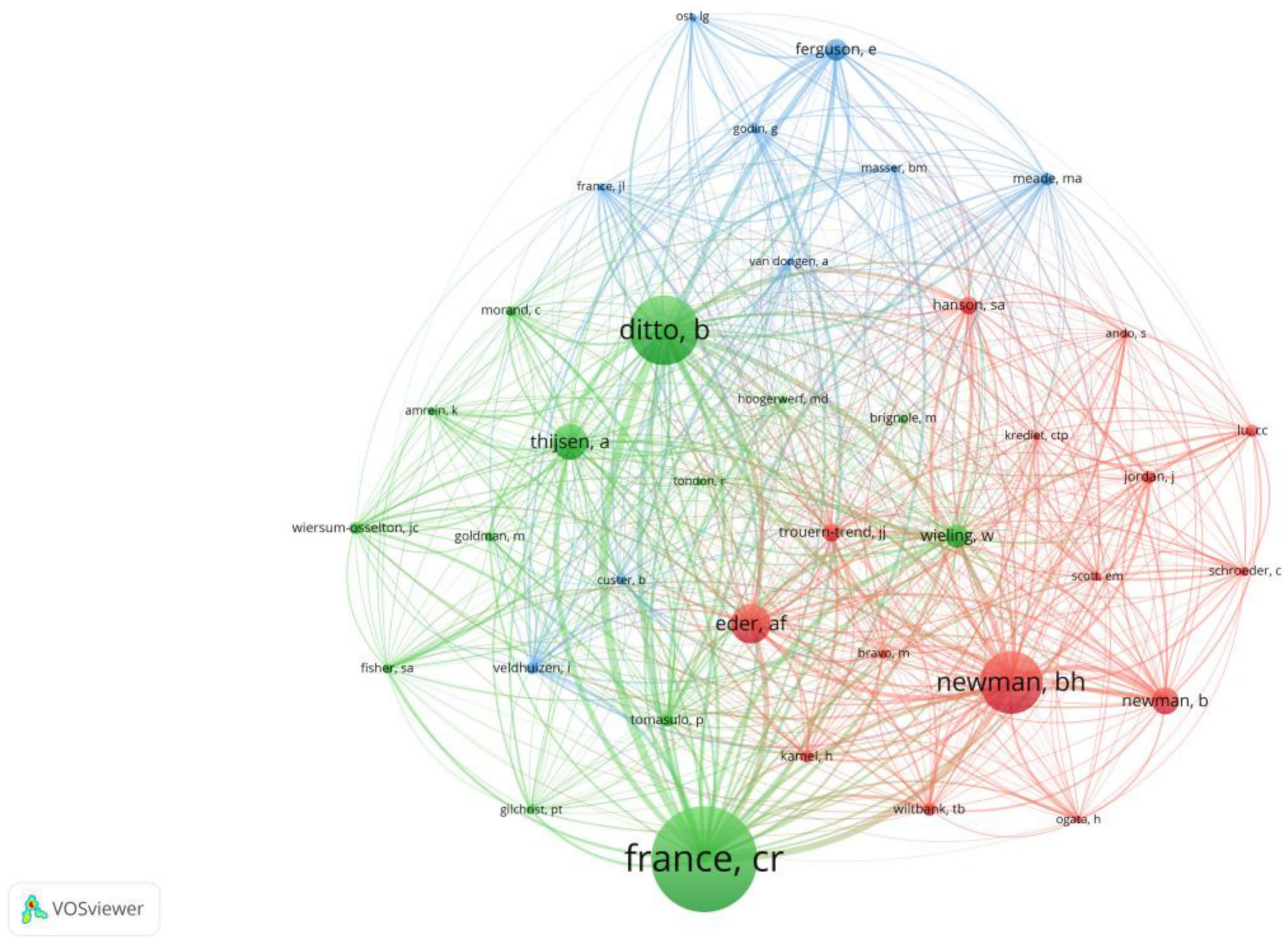
The visualization of co-cited authors on research of predonation hydration and VVR.

Among the 1,013 co-cited authors, France, CR received the highest citation frequency. The top three most cited authors were France, CR (*n* = 147), Ditto, B (*n* = 97), and Newman, BH (*n* = 87). Through VOSviewer analysis, we filtered authors with a minimum co-citation frequency of 10 and plotted the co-cited authorship map (Figure 6).

### Analysis of co-cited references

**Figure 7:**
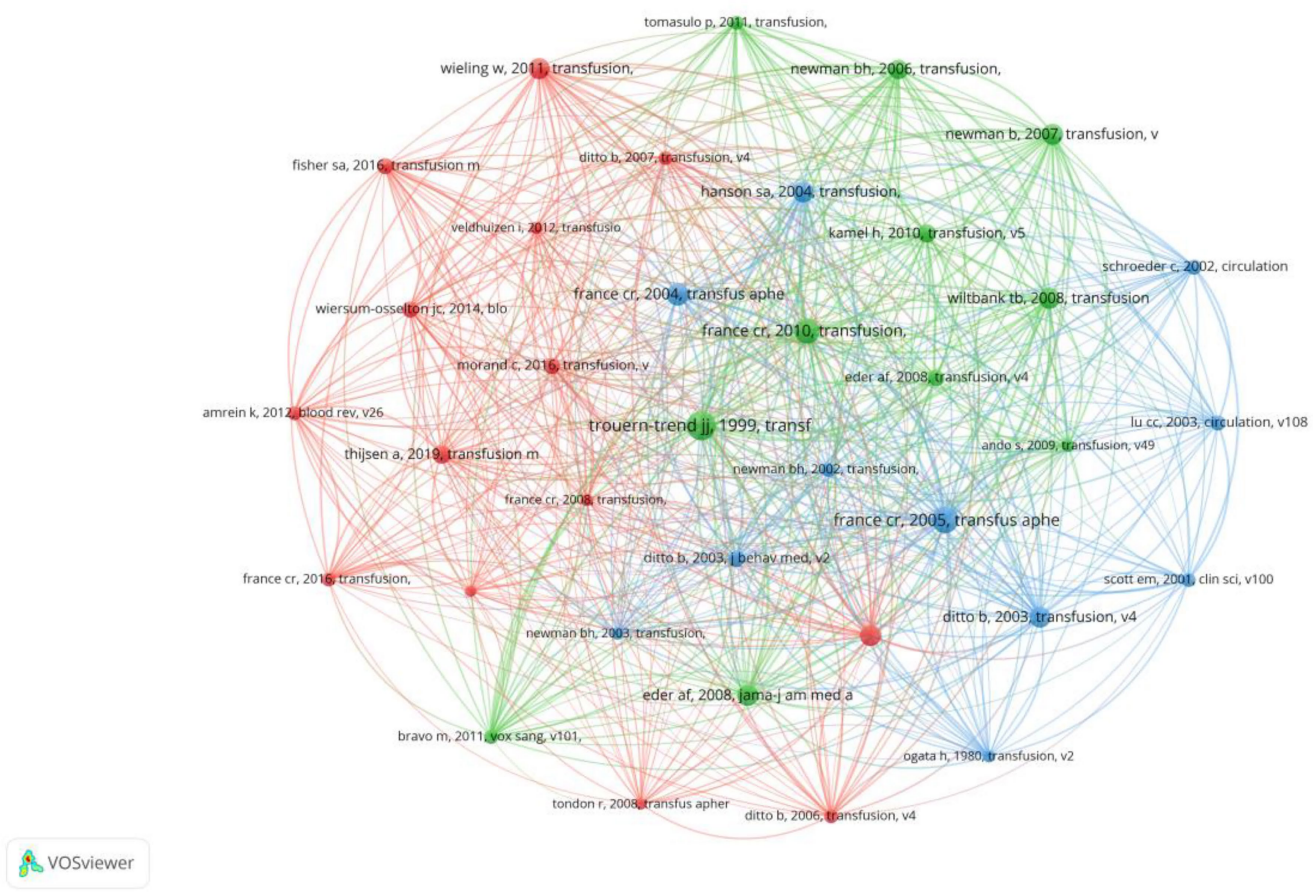
The visualization of co-cited references on research of predonation hydration and VVR.

The most cited references within a specific field reveal the impact of research. Among the top 10 co-cited references, citations ranged from 25 (Trouern-Trend JJ, 1999, Transfusion, v39, p316, doi: 10.1046/j.1537-2995.1999.39399219291.x) to 18, with an average of around 20 citations each (Table 4). We used VOSviewer to select co-cited references with a frequency greater than or equal to 10 to construct the co-cited reference network (Figure 7).

**Table 4:** Top 10 co-cited references on research of predonation hydration and VVR.

Rank	Co-cited reference	Citations	Link strength
1	trouern-trend jj, 1999, transfusion, v39, p316, doi 10.1046/j.1537-2995.1999.39399219291.x	25	97
2	france cr, 2005, transfus apher sci, v33, p99, doi 10.1016/j.transci.2005.02.005	23	83
3	france cr, 2010, transfusion, v50, p1257, doi 10.1111/j.1537-2995.2009.02574.x	22	85
4	france cr, 2004, transfus apher sci, v30, p17, doi 10.1016/j.transci.2003.08.014	19	63
5	ditto b, 2003, transfusion, v43, p1269, doi 10.1046/j.1537-2995.2003.00488.x	18	72
6	eder af, 2008, jama-j am med assoc., v299, p2279, doi 10.1001/jama.299.19.2279	18	79
7	hanson sa, 2004, transfusion, v44, p924, doi 10.1111/j.1537-2995.2004.03426.x	18	70
8	newman b, 2007, transfusion, v47, p1524, doi 10.1111/j.1537-2995.2007.01293.x	18	83
9	wieling w, 2011, transfusion, v51, p2727, doi 10.1111/j.1537-2995.2011.03202.x	18	52
10	wiltbank tb, 2008, transfusion, v48, p1799, doi 10.1111/j.1537-2995.2008.01745.x	18	82

### Reference burst analysis

**Figure 8:**
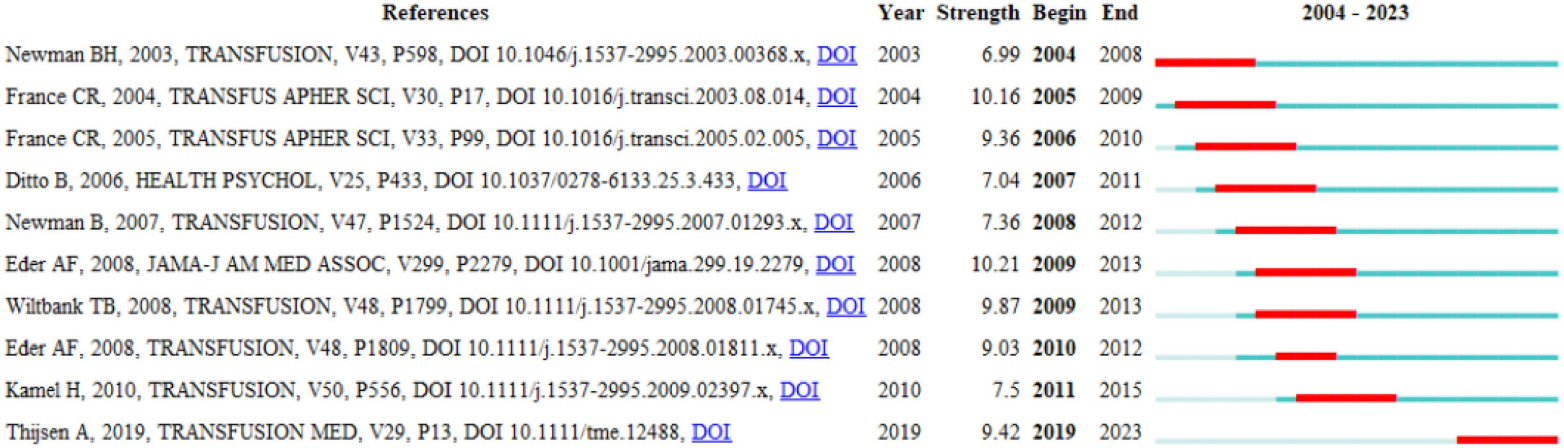
Top 10 references with the strongest citation bursts in the field of predonation hydration and VVR.

Reference burst refers to references frequently cited by scholars in a field during a certain period. Using CiteSpace, we identified 10 references with strong reference bursts (Figure 8). In the graph, the burst strength of these 10 references ranged from 6.99 to 10.21, with burst duration ranging from 2 to 5 years. Red bars represent strong bursts, with each bar representing 1 year. The burst of references in this study spanned from 2004 to 2023. The strongest burst of citations in the references was (Strength = 10.21) titled “Eder AF, 2008, JAMA-J AM MED ASSOC, V299, P2279, DOI: 10.1001/jama.299.19.2279,” with a burst period from 2009 to 2013.

### Hotspot and frontier analysis

**Figure 9:**
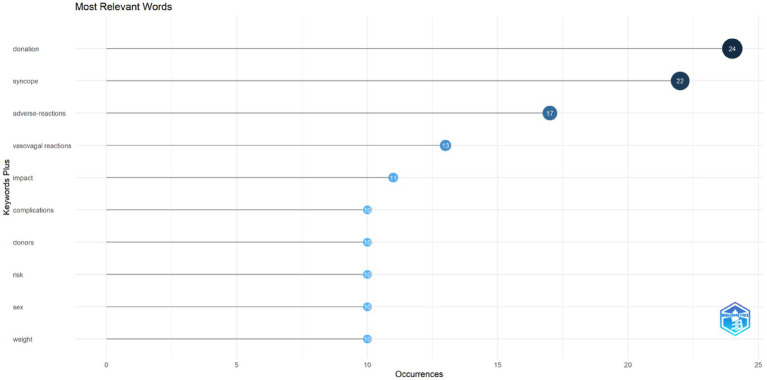
The visualization of most relevant words on research of predonation hydration and VVR.

**Figure 10:**
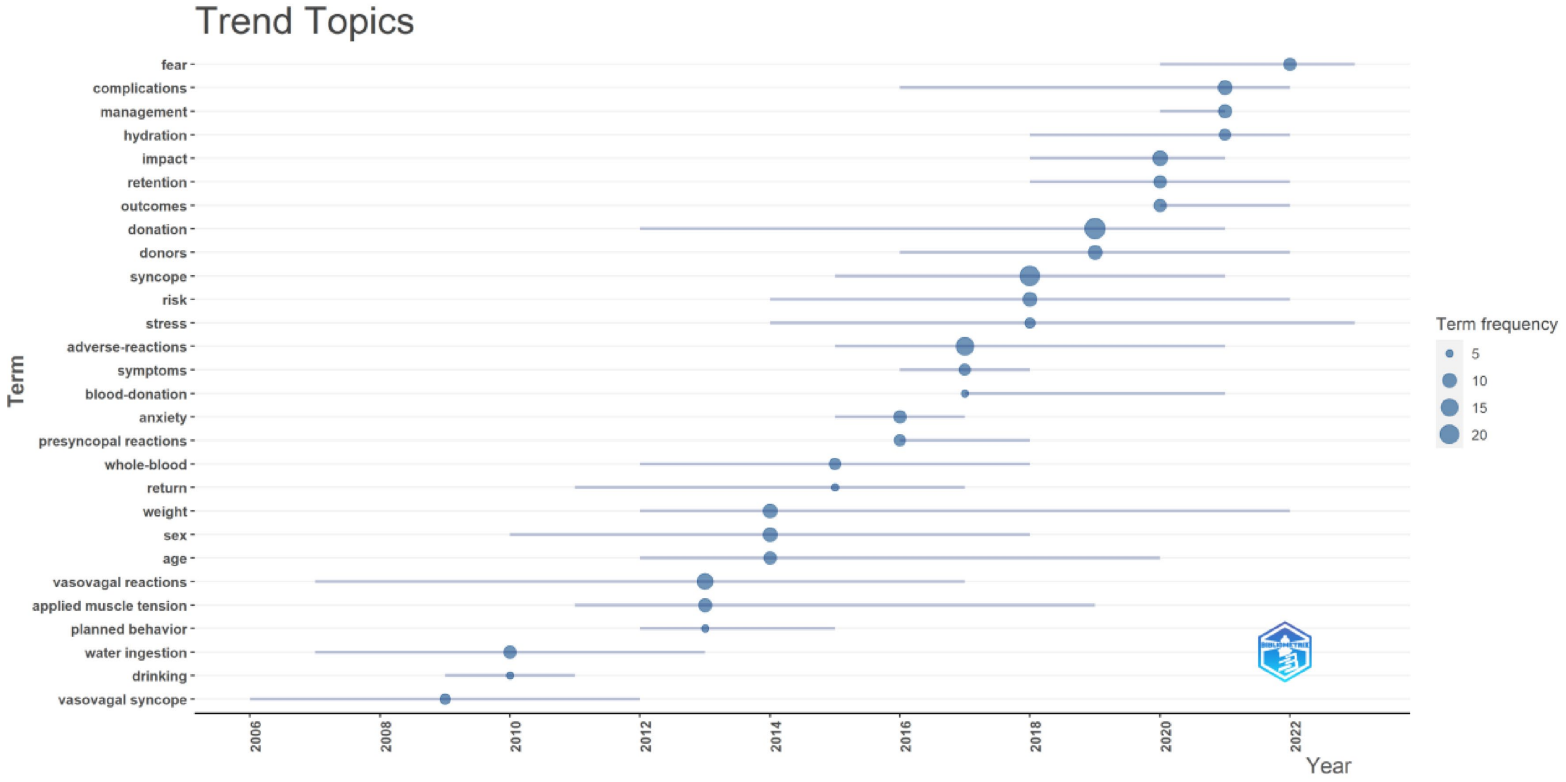
The visualization of trend topics on research of predonation hydration and VVR.

**Figure 11:**
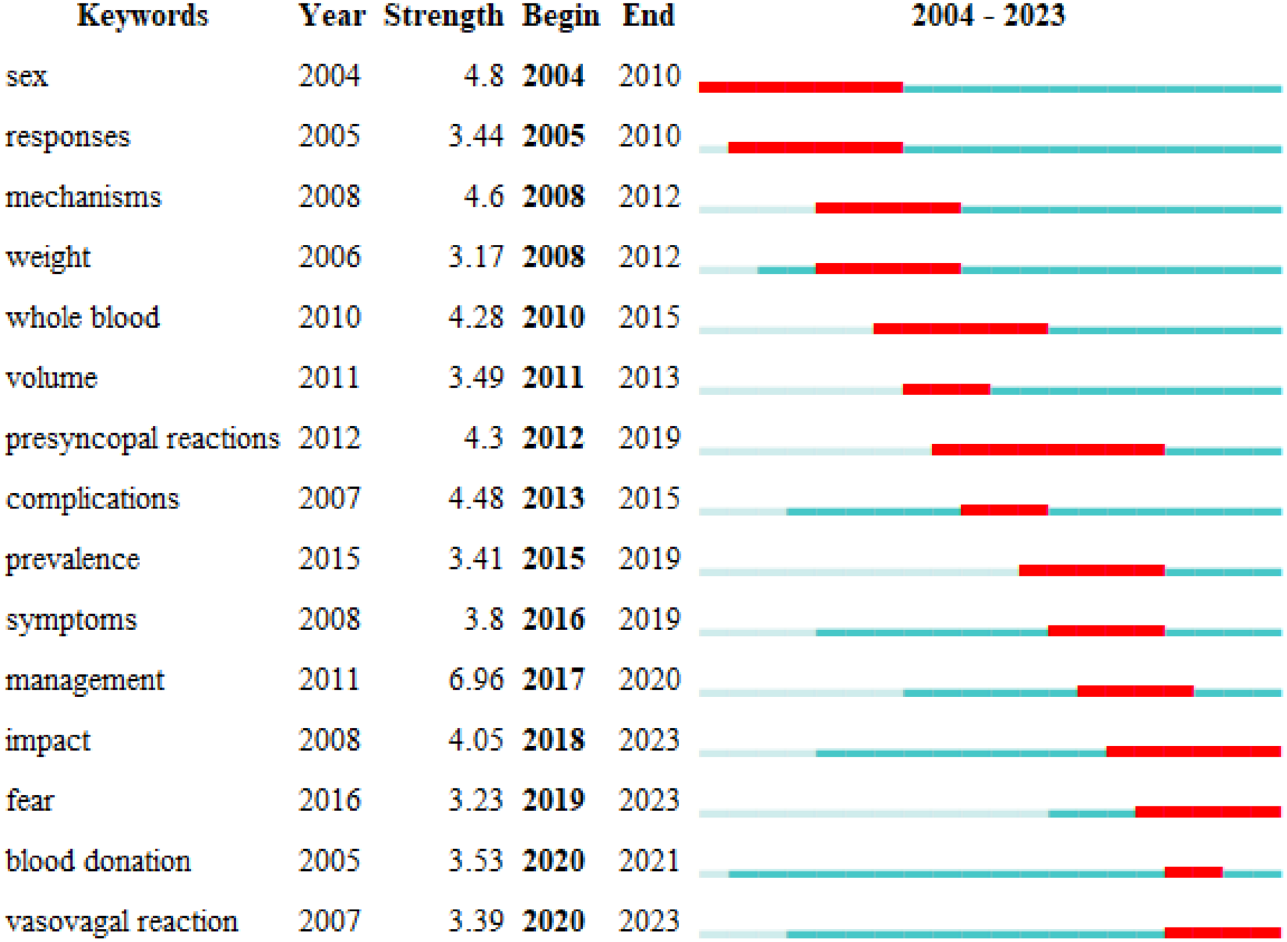
Top 15 keywords with the strongest citation bursts in the field of predonation hydration and VVR.

We conducted keyword co-occurrence analysis using bibliometrix. Among the top 10 high-frequency keywords shown in the graph (Figure 9), we found that terms such as “donation,” “syncope,” “adverse-reactions,” “vasovagal reactions,” “impact,” “complications,” “donors,” “risk,” “sex,” and “weight” appeared more than 10 times, indicating key research directions regarding predonation hydration and VVR. The thematic trend analysis of keywords revealed 28 main themes included in this study. Over the past 3 years (2021–2023), research trends primarily focused on “fear,” “complication,” “management,” and “hydration” (Figure 10). Keyword burst refers to keywords frequently used by scholars in a field during a certain period. We used CiteSpace to display the top 15 keywords (Figure 11). In this graph, the burst strength of these 15 keywords ranged from 3.17 to 6.96, with burst duration spanning 3 to 8 years. The keyword with the longest burst duration was “presyncopal reactions,” totaling 8 years. The keyword with the highest burst strength was “management” (Strength = 6.96).

## Discussion

This study conducts a bibliometric analysis of the literature on predonation hydration for preventing and reducing VVR over the past two decades. The results indicate a steady increase in the publication output related to predonation hydration for preventing and reducing VVR. Based on annual publication counts, the growth trend can be divided into slow and rapid stages. The slow growth stage occurred from 2004 to 2014, with fewer than 8 papers published annually. From 2015 to 2023, relevant research entered a rapid growth stage, with the number of papers published annually exceeding 17. Significant progress in publication output was observed in 2021, reaching 27 papers. This suggests that research on predonation hydration for preventing and reducing VVR may still be in an active and stable stage in the coming years. The underlying reasons behind this phenomenon may involve personalized approaches to predonation prevention and reduction of VVR gaining more attention. To provide more accurate and effective preventive measures for blood donors, more institutions are paying increased attention and support to this field, thus contributing to the high growth in recent years. In this area, the top 5 countries account for over half of the total publications (57.4%). The United States leads in publication output in this field and plays a significant role in international collaboration. Although the United States exhibits the highest publication output, only 2 of the top 5 institutions are from the United States, while China and Russia, ranking third in total publications, do not have any institution ranked in the top 5. Romania’s strong representation in our dataset (second only to the United States) likely reflects the concentrated activity of research groups at the University of Bucharest, which accounts for over one-third of the country’s output. Active cross-border collaborations, particularly with Western European partners, and national reporting practices that facilitate publication may also contribute to its disproportionately high bibliometric presence despite modest national blood donation volumes.

In terms of journal influence, impact factor, and JCR are effective indicators for evaluating journal impact. Among the top 5 journals, they are mostly JCR Q3 and Q4 journals. The journal types are mostly specialized (hematology journals), with no comprehensive journals. The related research is relatively limited, making it difficult to attract more scholars to study. Among them, *Transfusion* has the highest publication output. Core journals often bear the responsibility of publishing fundamental research findings in related fields. Therefore, for researchers newly entering this field, these journals can be primarily considered as the main reference direction. In addition, although China has made significant contributions in this field, ranking third in publication output, no Asian publishers are responsible for the top five journals. The influence of Asian journals in this field is relatively weak.

One of the primary objectives of this study is to address the scientific hotspots that researchers have widely focused on in the field of preventing (VVR during blood donation). Research hotspots can be analyzed from various perspectives, including publications, references, and keywords. Frequently cited literature reflects the core themes of a specific research area, which aids in identifying research hotspots. The top 10 publications in this study focus on factors influencing VVR, preventive measures for VVR, predictive indicators of VVR, and donor retention. Among the most cited publications, Trouern-Trend J. et al. found that even mild VVR during blood donation could affect donors’ willingness to donate blood again. Female donors, younger donors, first-time donors, and donors with low body weight have a higher incidence of VVR than other donors (34). Keywords represent the core content of studies, and the frequency of keywords indicates the impact of such terms in a specific field. These keywords represent another aspect of research hotspots. The most commonly used keywords are related to the components of “vasovagal reactions,” such as “syncope,” “influencing factors,” “risk factors,” “gender,” “weight,” “adverse reactions,” and “donors,” all of which rank in the top 10 in terms of frequency. The degree of imputability between donation and adverse events, including VVR, is defined in the ISBT/IHN Standard for Surveillance of Complications Related to Blood Donation, which provides internationally recognized categories (‘definite,’ ‘probable,’ ‘possible,’ ‘unlikely,’ and ‘excluded’). While many hemovigilance-based studies in our dataset used national reporting systems aligned with these standards, others—especially older or single-center studies—did not specify the use of formal imputability criteria. This variability underscores the importance of harmonizing definitions to ensure comparability of data across studies and countries.

Reference burst analysis is a method provided by CiteSpace to demonstrate references that have undergone significant changes during a specific period. In reference analysis, citation strength is an indicator that reveals the attraction intensity of a reference. The higher the citation strength, the greater the attention and discussion it has received. The reference burst analysis time represents an indicator of the continuous citation of a reference during a certain period. The reference with the highest citation strength comes from Eder AF et al., whose research primarily focuses on minors aged 16 and 17 donating blood in the United States. Compared to older donors, minors aged 16 and 17 have a higher incidence of blood donation-related complications and injuries (35). Therefore, recruiting and retaining young blood donors require more preventive measures. Keyword burst analysis is also a method provided by CiteSpace to demonstrate keywords that have undergone significant changes during a specific period. Through keyword burst analysis, another method for identifying research hotspots can be provided. This study found that the longest-lasting keyword in nearly 20 years of research is “presyncopal reactions,” spanning from 2012 to 2019, covering an 8-year period. Recent research has mainly focused on “donor management,” “donor impact,” “donor psychological factors,” and “mechanisms of vasovagal reactions.”

In terms of blood donor management, research indicates that the incidence of VVR during whole blood donation ranges from 1.4 to 7% for presyncopal reactions and from 0.1 to 0.5% for syncope (36). Conversely, another report from 201 blood centers in China for the years 2012, 2013, and 2014 showed VVR incidence rates for whole blood donation of 0.44, 0.45, and 0.53%, respectively, which are much lower than those reported in previous studies (37). Most studies in our dataset reported VVR occurrence as a single outcome without differentiating severity. However, a subset of randomized and observational studies explicitly graded reactions and indicated that predonation hydration reduced not only the overall incidence but also the severity of VVRs, with fewer episodes of frank syncope. The significant discrepancy in data may be due to the fact that many blood donors who experience VVR after leaving the donation site may not report it to the blood centers. Therefore, it is necessary to develop a predictive model for VVR based on existing data, categorizing blood donors according to demographic information and intervening specifically for those at higher risk of VVR. Additionally, post-donation follow-up should be conducted for high-risk donors to assess the effectiveness of intervention measures. We also note that prolonged fasting (long intervals since the last meal) is associated with increased vasovagal risk in blood donors, with observational data identifying ‘time after eating ≥4 h’ as an independent predictor of VVR. Accordingly, donor education should emphasize a light meal and adequate hydration before donation (37).

Regarding the impact and psychological factors of blood donors, individual differences should be a focal point. It is important to examine how blood donors of different ages, genders, and health statuses respond to the intervention of predonation hydration. Research reports have indicated that female blood donors are more likely to experience VVR compared to males. Through data analysis and comparison, developing and evaluating hydration protocols before donation tailored to different genders and weights can help reduce the incidence of VVR during blood donation. Considering potential differences in muscle mass, physical strength, and hormone levels between males and females, a multifaceted approach should be taken in research. Although several studies report a protective effect of predonation hydration, others have found no significant benefit in reducing vasovagal reactions or presyncopal symptoms (38). Such conflicting results may be attributable to variations in study design, donor demographics, hydration protocols, and definitions of adverse events. Although most studies in our bibliometric dataset reported only the occurrence of VVR, a subset also graded reaction severity. These studies suggest that predonation hydration may reduce not only the incidence but also the severity of vasovagal reactions, including fewer episodes of frank syncope or prolonged post-donation recovery (28, 38). Such findings are clinically important, as even when prevention is incomplete, reducing severity can improve donor safety, comfort, and willingness to return. Future primary research and meta-analyses should systematically include severity grading to better inform donor management protocols. Considering the strong link between adverse donation reactions and reduced donor return rates, hydration has important implications for donor fidelization. The available evidence, including multiple randomized trials, indicates that when hydration is applied with adequate volume and timing, it can lower both the incidence and severity of vasovagal events. This reduction in negative donor experiences is likely to enhance willingness to donate again. While conflicting results exist, these appear to stem largely from heterogeneity in hydration protocols and study populations rather than a null effect. Therefore, from a practical standpoint, hydration represents a low-cost, non-invasive, and easily implementable measure that, if standardized, could make a meaningful contribution to donor retention strategies.

Our bibliometric review found that only a subset of primary studies explicitly reported the hydration protocol details, most commonly volumes of 330 mL or 500 mL of water consumed approximately 10–20 min before phlebotomy. Several trials compared these volumes, with mixed results—some reporting reduced presyncopal reactions with larger volumes, while others observed no significant difference. Timing between ingestion and donation also varied, generally ranging from 5 to 30 min. These inconsistencies highlight the need for future intervention trials to adopt standardized hydration protocols, specifying both volume and timing, to allow for direct comparison across studies. Across the studies included in this bibliometric analysis, no adverse effects of predonation hydration were reported. Water intake is generally regarded as a safe, low-cost, and non-invasive intervention. The only theoretical risk would be related to the quality of the water provided; for example, if water supplies were microbiologically contaminated. However, in all clinical trials and institutional practices identified, hydration was implemented with safe drinking water, and no complications were documented. Thus, while the evidence base supports hydration as a safe strategy, maintaining high standards of water quality remains essential for donor safety.

In terms of VVR mechanisms, research reports suggest that VVR primarily occurs due to vasodilation-induced hypotension. In this process, individuals go through four stages: (1) stable stage, (2) circulatory unstable stage, (3) hypotension stage, and (4) recovery stage. Therefore, reducing the hypotensive state of blood donors, especially during the circulatory unstable stage, becomes crucial. The study also indicates that nearly all adults experience a decrease in cardiac output, but only younger individuals gradually reduce vascular tone (9). In routine blood collection, strategies to prevent blood donors from entering the second stage can greatly reduce the probability of VVR occurrence.

In interpreting our findings, it is important to consider the potential influence of national haemovigilance system efficiency on the bibliometric patterns observed (39). Countries such as the United States, Australia, and parts of Europe maintain mature, centralized haemovigilance systems aligned with ISBT/IHN definitions, ensuring high reporting completeness and standardized classification of adverse events. These systems may facilitate both early detection of vasovagal reaction patterns and the generation of robust research output. In contrast, some countries represented in our dataset, despite substantial donor volumes, rely on decentralized or voluntary reporting, with limited adoption of formal imputability criteria. This can result in underreporting or heterogeneity in recorded events, potentially influencing the apparent incidence of VVR and the subsequent research emphasis. Therefore, our results should be interpreted with the recognition that bibliometric productivity may partially reflect differences in haemovigilance infrastructure and reporting efficiency, rather than solely research interest or burden of adverse events.

## Conclusion

In summary, research on VVR and predonation hydration is increasingly gaining attention, with related studies showing a steady upward trend. New directions for future research hotspots include: 1. Identification of risk factors and influencing factors of VVR. 2. Strategies for preventing and managing VVR. 3. Understanding the symptoms associated with VVR. Through the analysis of the results mentioned above, researchers can conduct more precise and comprehensive studies in the field of VVR and predonation hydration. In particular, future work should evaluate standardized pre-donation hydration protocols—specifying the optimal volume (e.g., 330 mL vs. 500 mL) and the ideal time interval before donation—to ensure consistent preventive strategies and comparability of outcomes.

### Strengths and weaknesses

This study has three distinct strengths. Firstly, it marks the first systematic analysis of the relationship between predonation hydration and VVR occurrence using bibliometric methods, providing detailed and accurate insights for scholars in this field. Secondly, the use of visualization bibliometric software such as VOSviewer, R package “bibliometrix,” and CiteSpace ensures more objective conclusions compared to traditional literature reviews. Lastly, bibliometric analysis enables the prediction of future research trends and frontiers in this field.

However, this study also has several limitations. Firstly, the data sources were limited to the WoSCC database, excluding other databases such as PubMed and CNKI. Secondly, the language restriction to English omitted non-English literature, potentially excluding valuable studies. Lastly, due to time constraints, the study commenced at the end of 2023, precluding the inclusion of research conducted in early 2024.

## Data Availability

The original contributions presented in the study are included in the article/supplementary material, further inquiries can be directed to the corresponding author.
